# 

**Published:** 1883-03

**Authors:** 


In consequence of the recent fire in our Bindery and the increased demand for The Independent Practitioner, we should feel obliged if those who have received sample copies—February, 1883—and do not wish to subscribe would return the same to the Columbia Publishing Co.

Vol. IV. MARCH, 1883. No. 3.

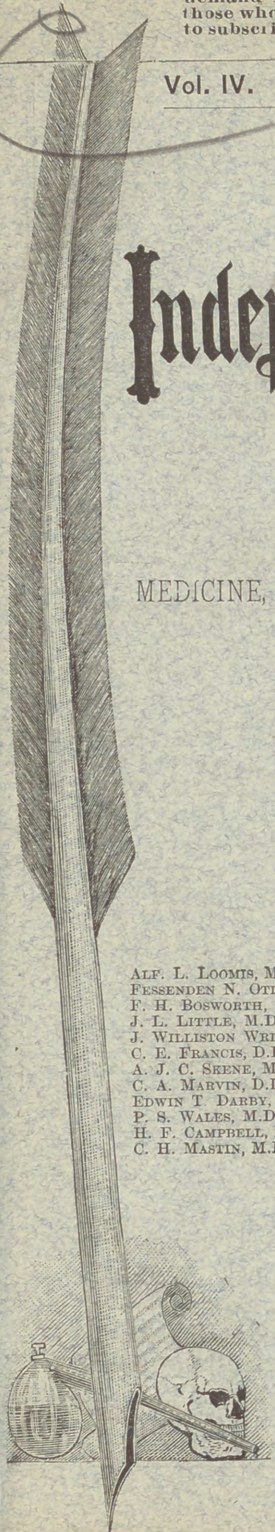

S.G.O. INDEXED

THE

### Independent Practitioner:

A MONTHLY JOURNAL,

DEVOTED TO

MEDICINE, SURGERY, OBSTETRICS, DENTISTRY, PATHOLOGY AND POPULAR SCIENCE.

EDITORS:

LEIGH H. HUNT, B.Sc., M.D.

W. C. BARRETT, D.D.S., M.D.

NEW YORK:
*THE COLUMBIA PUBLISHING CO.,*
42 DUANE STREET.

$2.50 Per Annum in Advance. - - Single Copies, 25 Cents.

Entered at the Post-Office, New York City, as Second-Class matter.

Read Advertisement on Page 12.

